# Exosome-Derived LncRNAs in Lung Cancer

**DOI:** 10.3389/fonc.2020.01728

**Published:** 2020-09-23

**Authors:** Tao Fan, Nan Sun, Jie He

**Affiliations:** ^1^Department of Oncology, Renmin Hospital of Wuhan University, Wuhan, China; ^2^Department of Thoracic Surgery, National Cancer Center, National Clinical Research Center for Cancer, Cancer Hospital, Chinese Academy of Medical Sciences, Peking Union Medical College, Beijing, China

**Keywords:** exosome, lncRNA, lung cancer, biomarkers, intercellular communication

## Abstract

As extracellular vesicles, exosomes are released from most cells to perform cell–cell communication. Recent studies have shown that exosomes could be released into tumor microenvironment and blood to promote tumor progression through packaging and transmitting various bioactive molecules, such as cholesterol, proteins, lipids, miRNAs, mRNAs, and long non-coding RNAs (lncRNAs) to distant cells. LncRNAs have emerged as a major class of non-coding transcripts. A lot of LncRNAs have been discovered during the past few years of research on genomics. They have been proven to participate in various biological functions and disease processes through multiple mechanisms. In this review, we analyzed the role of exosome-derived lncRNAs in lung carcinogenesis and metastasis. We also highlight opportunities for the clinical potential of exosomes with specific lncRNAs as biomarkers and therapeutic intervention in lung cancer.

## Introduction

Lung cancer is still the most common malignancy with the highest morbidity and mortality. In China, it has been estimated that 733,300 new cases of lung cancer were diagnosed and 610,200 cases died in 2015 (1). In America, approximately 228,150 people were firstly diagnosed with lung cancer and 142,670 patients with lung cancer died in 2019 (2). High mortality rate occurred in lung cancer patients mainly due to late-stage diagnosis of the disease (3). Patients often go to the hospital with symptoms of chest distress, hemoptysis, or systemic involvement. The 5-year survival rate of lung cancer is as low as 4% for patient with distant metastases (4), and the overall 5-year survival rate is only 24%. In view of this, it is of great importance to clarify the mechanism of lung cancer metastasis, find relevant biomarkers for early diagnosis, and treat patients with precision therapy.

Recent studies have shown that exosomes play an important role in the metastasis of lung cancer cells (5–8). Characterized by inflammation, angiogenesis, immunosuppression, and organotropism, pre-metastatic niche is used to spread tumor cells (9–11). It has been confirmed that exosomes secreted by primary tumor site possibly contribute to the establishment of premetastatic niche (12–14). Various bioactive molecules, such as proteins, RNAs, cholesterol, etc., are encapsulated by exosomes and transported to adjacent cells or distant tissues to optimize tumor microenvironment (15–17). LncRNAs are emerging as epigenetic regulators affecting transcription and playing a key role in human health (18–21). In addition to directly regulating intracellular biological activities, lncRNAs can also be detached from the surface of primary cells in the form of exosomes, which were subsequently transmitted to adjacent cells or distant organs through circulatory system (22–24). Exosome-transmitted lncRNAs have been detected in both blood and tumor tissues in lung cancer (25–27). In this review, we highlight the exosomal lncRNAs in lung cancer and summarize the potential biomarkers and mechanisms of exosome-derived lncRNAs.

## Exosome Biology and Function

**FIGURE 1:**
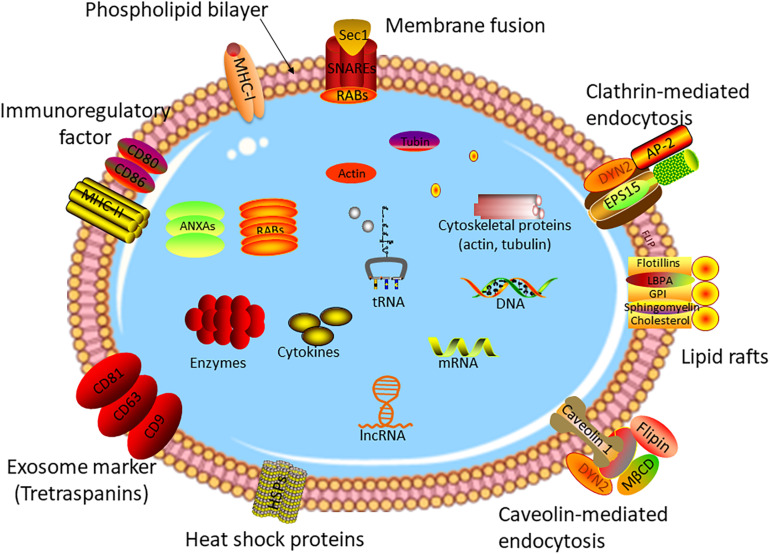
Structure of exosome. Exosomes are small vesicles with a double-membrane structure. The phospholipid bilayer is embedded with many transmembrane proteins, marker proteins, and receptors, such as heat chock proteins, lipid rafts, immune-regulatory molecules, cytoskeleton proteins, membrane fusion proteins, etc. Exosomes consisted of DNA, RNAs, cytokines, enzymes, and other bioactive molecules, which are transmitted from original cells to target cells.

As one of three types of extracellular vesicles, exosomes have a diameter of 40–150 nm, which can be secreted by a vast majority of cells (28–30). The formation mechanism of exosomes is different from other types of vesicles. For the first step, the plasma membrane buds inward to form early endosomes (membrane-bound vacuoles) (31, 32). After undergoing several changes, the late endosomes named multivesicular bodies (MVBs) form with the membrane-bound vacuoles budding inward and pinching off to shape intraluminal vesicles (ILVs) (33, 34). Subsequently, the ILV-loaded MVBs release into extracellular space to act as exosomes or fuse with lysosomes to degrade the ILV contents (35–37). Vesicles with exosome-like structures were discovered by (38), while the true definition of exosomes was in 1983 (39). During the past decade, with the in-depth study of exosomes, it has been found that exosomes are involved in the development and prognosis of various diseases (40–44), and especially, play an irreplaceable role in tumor invasion (45), metastasis (46), and progression (47). Exosomes contain a variety of bioactive molecules, including proteins, RNAs, cholesterol, and lipids (24, 48–52). Figure 1 shows the structure and contents of exosomes. The contents of exosomes are the key to the biological function of exosomes in the pathophysiological process. For example, plasma exosomal proteins derived from endothelial cells play an important role in small cerebral vascular disease caused by Alzheimer’s disease (53). Exosomes shuttling high levels of microRNA-221-3p promote the resistance of breast cancer cells to adriamycin by targeting PIK3R1 (54).

## Exosomes Loaded With Cargos and Secreted by Donor Cells

The current mainstream view is that cell membrane invaginates and internalizes extracellular ligands and particles to form nascent exosomes, which originate from ILVs. ILVs fused with the serous membrane to form multivesicular endosomes (MVEs) with packaging various bioactive molecules. Endosomal sorting complex required for transport (ESCRT) is the key molecular mechanism in MVE membrane shaping and scissing, and as a primary driver, ESCRT enables cargo selection and ILV budding (55–57). Mediated by Rab family and soluble *N*-ethyl-maleimide-sensitive factor attachment protein receptor (SNARE) family, mature exosomes are released by donor cells (58, 59). The Rab families are small GTPases that control the transport and secretion of intracellular secretory MVBs by moving the cytoskeleton and positioning the vesicles in the cytoplasmic membrane (60, 61). More than 70 subtypes have been found with the in-depth study of Rab GTPases, which were located on different cell membrane surfaces. MVE traffic regulated by Rab GTPases affects physiology and disease. For example, Rab5 is an endosome organizer to transfer dipeptidyl peptidase 4 (DPPIV) and bile salt export pump (BSEP) to the bile canaliculi (62). SNARE was originally discovered in bovine brain extract, which was verified to be a key molecule in the release of neurotransmitters (63). SNARE complex assembly was initiated by the docking of GTPases and membrane (64). SNARE complex drives membrane fusion through Sec3 interacting with Sso2 (target membrane protein of SNARE) (65). Located on the surface of secretory vesicles, activated RAL-1 recruits SYX-5 at the surface of plasma membrane to promote fusion of MVBs, and further to promote secretion of exosomes (66).

## Exosome Uptake and Function

Exosome uptake is a very complex biological process. Exosomes containing bioactive molecules bind to acceptor cells directly by cell membrane fusion, endocytosis, and cell-specific uptake (67). Recipient cells identify and capture exosomes probably depending on their size or surface biomolecules (67). Eguchi and Yang have demonstrated that clathrin-mediated endocytosis and macropinocytosis are effective means to promote exosomes to be ingested by target cells (68, 69). Caveola-related endocytosis was also detected in EB virus-infected cells (70). Lipid is the main component of cell membrane. As microdomains interspersed in cell membrane, lipid rafts play a key role in the passage of molecules across cell membranes. It has been proven that some lipid rafts exist in caveolin-1-mediated invaginations or planar regions within cell membrane, and these regions are rich in flotillin proteins (67, 71, 72). So, lipid rafts may be involved in the process of internalizing exosomes through flotillin-mediated endocytosis.

The function of exosomes is complex and worth exploring. It is interesting to note that the target cell phenotype may be changed by exosomes (73). Exosomal miR-21 triggered phenotypic changes of hypoxic oral squamous cell carcinoma to promote cancer metastasis in a hypoxia-inducible factor-1-dependent manner (74). Melanoma-derived exosomes, composed of tumor microenvironment oncoproteins, induced bone marrow progenitor cells to a prometastatic phenotype (75). In addition, exosomes have been emerging as ideal biomarkers for containing active molecules of special significance. For example, Zhu and colleagues found that exosome-transmitted transfer ribonucleic acid (tRNA)-derived small RNAs, such as tRNA-ValTAC-3 and tRNA-ValAAC-5, were dramatically elevated in exosomes separated from the liver cancer patient’s blood, which indicated that exosomal tsRNA could function as a potential biomarker for cancer diagnosis (76). LncRNA growth arrest-specific 5 (GAS5) mediated by exosomes was also identified as a promising serum-based biomarker for early non-small-cell lung cancer (NSCLC) diagnosis (77). It is widely proven that some exosomal DNAs, RNAs, proteins, and other bioactive molecules are emerging as critical and ideal non-invasive biomarkers for various benign and malignant diseases (78–82).

As mentioned before, exosomes can encapsulate bioactive molecules and then intelligently identify the recipient cells to achieve information transmission. Exosomes loaded with special drugs to treat diseases gradually attracted the attention of scholars. The main advantages of exosomes in drug delivery are the cell-targeted therapy, non-toxicity, and non-immunogenicity. Zou and colleagues synthesized Apt-Exos, which combined the natural delivery advantages of exosomes and superiority of aptamers of molecular recognition to provide an ideal delivery platform for cancer theranostics (83). It has been confirmed that miRNA cargo in exosomes derived from administered cells has therapeutic effects in brain repair and recovery after neurological injury (84). As a novel therapeutic approach, drugs transmitted by exosomes has entered the preclinical research phase (85). In recent years, exosome-related nanovesicles have been used to the precise treatment of diseases (86–88).

## Characteristics of LncRNAs

**FIGURE 2:**
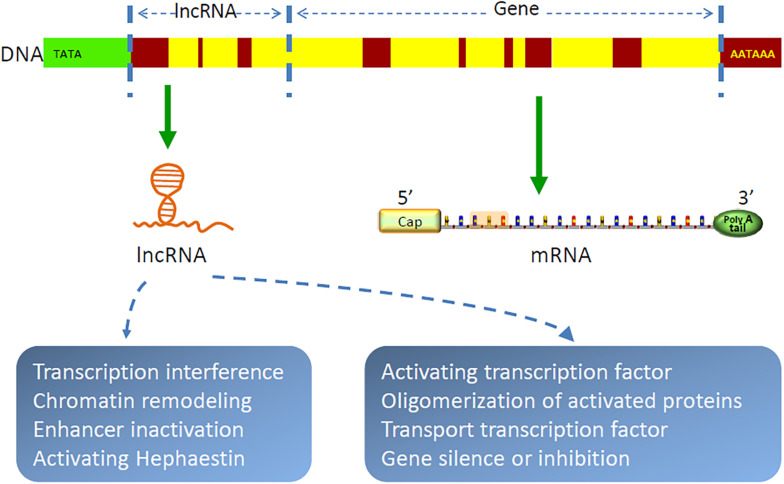
Structure and function of lncRNAs. LncRNAs are by-products of RNA polymerase II transcription, which function as chromatin remodeling, regulating gene transcription and translation, etc.

Gene transcription in organisms is a complex and orderly process. Most genome sequences are transcribed into coding RNAs or non-coding RNAs (89). As a class of non-coding RNAs, lncRNAs are recently identified as a group of long RNA transcripts with no apparent protein-coding function (90), which are divided into five categories, as follows: intronic, sense, antisense, intergenic, and bidirectional. With the implementation of the human genome project and the development of molecular biology, lncRNAs have been increasingly discovered (91–93). They are evolutionarily conservative and contain relatively few exons (94). It has been demonstrated that lncRNAs are by-products of RNA polymerase II transcription, which can mediate chromosome remodeling and prevent the recruitment of chromatin modifiers to affect gene expression (95–100). In cytoplasm, lncRNAs are largely reported to sponge miRNAs to regulate gene expression (101–105). For example, FOXF1 adjacent non-coding developmental regulatory RNA (FENDRR) was identified as downregulated in lung cancer, and high expression of which could suppress the progression of NSCLC by regulating miRNA-761/TIM2 axis (106). CDKN2B antisense RNA 1 (CDKN2B-AS1) was proven to be highly expressed in NSCLC (107), which promoted cancer cell proliferation and migration (108). LncRNAs also form complementary double chains with transcripts of protein-coding genes and produce endogenous siRNAs under the action of dicer enzyme to regulate gene expression level (109). In addition, lncRNAs can bind with proteins, regulate protein functions, and participate in protein degradation (110–112). Peng and colleagues found that lncRNA rhabdomyosarcoma 2-associated transcript (RMST) increased the DNA methyltransferases (DNMT3B) stability by promoting the interaction between RNA-binding protein HuR and DNMT3B 3’ UTR (113). Recent studies have shown that lncRNAs are also involved in autophagy regulation and m(6)A methylation (111, 114–117). In general, lncRNAs are involved in all aspects of life for their complex functions of chromatin modification, transcriptional regulation, pre-mRNA processing and splicing, RNA stability modulation, etc (118–121). Figure 2 shows the structure and function of lncRNA. Furthermore, as bioactive molecules, lncRNAs can be transmitted by exosomes to target cells or organs to exhibit their biological functions (23, 24).

## Lung Cancer-Related Exosomal LncRNAs

Although the free exosome database (ExoCarta) was launched in 2009, the first exosomal lncRNA in lung cancer was reported by Wang and colleagues in 2016. Wang and colleagues verified the new mechanisms that lung cancer cell-derived exosomes could regulate mesenchymal stem cells by transmitting lncRNAs (122). In recent years, some definite lncRNAs are discovered in exosomes secreted from lung cancer cells, which affect tumor progression, metastasis, and invasion. The latest researches concerning exosomal lncRNAs and lung cancer are summarized in Table 1.

**TABLE 1:** Exosomal lncRNAs in lung cancer.

LncRNAs	Exosome function	Origin of exosomes	Sample collection	Recipient cells	References
H19	Promotes gefitinib/erlotinib resistance	HCC827, HCC4006, A549	Blood, culture medium	Non-small cell lung cancer	(145, 146)
GAS5	Biomarker	NSCLC cells	Blood	/	(77)
GAS5	Promotes tumor angiogenesis	Lung cancer cells	Culture medium	Human umbilical vein endothelial cells	(133)
MALAT 1	Promotes cell proliferation and migration	NSCLC cells	Blood	A549, H1299	(25)
RP11-838N2.4	Promotes erlotinib resistance	NSCLC cells	Blood	HCC827, HCC4006	(147)
DLX6-AS1	Biomarker	NSCLC cells	Blood	/	(154)
/	Regulate silicosis	Lung tissue of silica-exposed rats	/	/	(155)
RP11-397D12.4, AC007403.1, and ERICH1-AS1	Biomarker	NSCLC cells	Blood	/	(156)
SOX2-OT	Biomarker	LUSC cells	Blood	/	(157)
/	Changes microenvironment	A549 cells	Culture media	Mesenchymal stem cells	(122)
HOTAIR	Promotes cell proliferation, migration, and invasion	Lung cancer cells	Blood	A549 and H1299	(138)
MSTRG.292666.16	Promotes osimertinib resistance	NSCLC cells, H1975 cells	Blood	H1975 cells	(148)
MRPL23-AS1	Creates a premetastatic microenvironment	SACC cells	Culture medium	HPMECs	(139)
UCA1	Promotes gefitinib resistance	NSCLC cells, HCC827, PC9	Blood, culture media	HCC827, PC9	(149)
UFC1	Promotes cell proliferation, migration, and invasion	NSCLC cells	Blood, culture media	A549, H1299	(158)

*“/” represent not explicit or mentioned; *NSCLC*, non-small cell lung cancer; *LUSC*, lung squamous cell carcinoma; *SACC*, salivary adenoid cystic carcinoma; *HCC827/HCC4006/H1975/A549/H1299/PC9*, lung cancer cell lines; *HPMECs*, human pulmonary microvascular endothelial cells.*

## Exosome-Derived LncRNAs in Lung Cancer Progress

Cargos carried by exosomes are involved in all stages of tumor progression. In the process of tumor progression, the most important molecular mechanism is that tumor cells adhere to the stroma, further migrate to blood, reach premetastatic niches, establish new tumor lesions, and achieve tumor metastasis. It is reported that exosomal lncRNAs promote tumor progression by facilitating tumor premetastatic niche formation (73, 123, 124). Furthermore, epithelial to mesenchymal transition (EMT) is also an important mechanism of tumorigenesis. EMT promotes tumor cells to escape from the original tumor site to form new metastases (125). Several studies have shown that tumor-derived exosomal lncRNAs play vital roles in regulating EMT (126–128).

**FIGURE 3:**
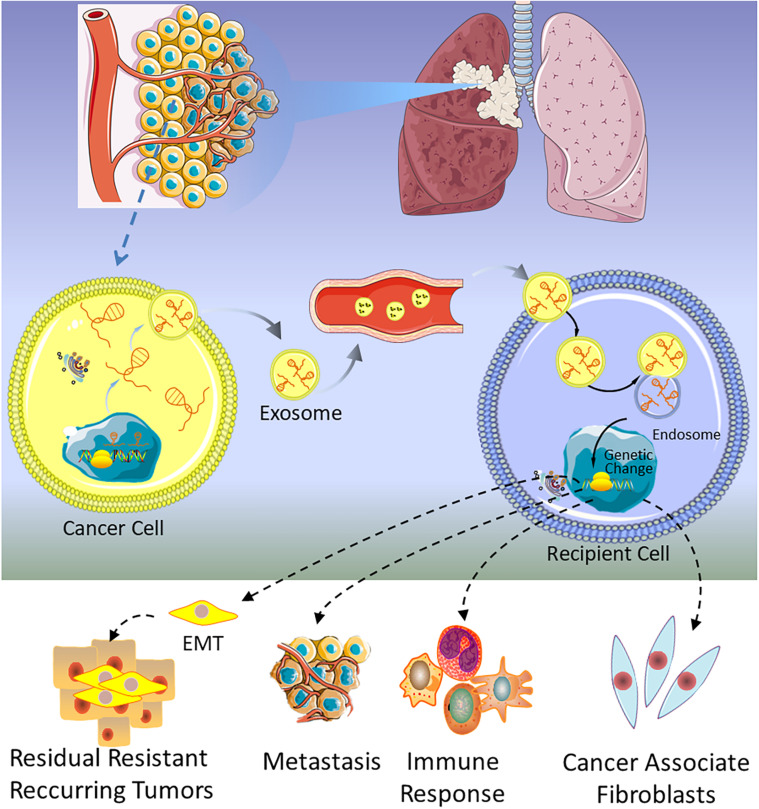
A schematic of the function of exosomal lncRNAs in lung cancer.

The first lncRNA found to be involved in lung cancer metastases was metastasis-associated lung adenocarcinoma transcript 1 (MALAT1) (129), and subsequently, Zhang and colleagues found that exosomal MALAT1 was highly expressed in NSCLC patients’ serum, which accelerated tumor migration and proliferation by suppressing cell apoptosis and shorting cell cycle (25). These studies suggest that exosomal MALAT1 may act as a non-invasive biomarker for diagnosis of NSCLC or be a promising therapeutic target for NSCLC. At the same time, a similar mechanism of exosomal MALAT1 in many other malignancies is also verified (130–132). As shown in Table 1, several lncRNAs have been discovered in lung cancer, which are involved in the progression, metastasis, invasion, and proliferation of tumor. For example, GAS5 has been proven to be a potential therapeutic target for lung cancer by inhibiting angiogenesis (133). Lung cancer-derived exosomal GAS5 regulates the apoptosis, proliferation, and tube formation of human umbilical vein endothelial cells (HUVECs) (133). Located on the cytogenetic band 12q13.13, homeobox transcript antisense intergenic RNA (HOTAIR) gene has five transcripts, which are all identified as lncRNAs. The expression level of HOTAIR was significantly increased in patients with (chronic obstructive pulmonary disease) COPD or lung cancer, especially in patients with advanced stage of the tumor (134, 135). Exosomal HOTAIR was first identified from serum of patients with glioblastoma multiforme (136), and then it was found to be highly expressed in bronchoalveolar lavage (BAL) of smokers, NSCLC, and healthy patients (137). NSCLC cell-derived exosomal HOTAIR promoted cancer cell proliferation, migration, and invasion by sequestrating miRNA-203 (138). Unlike the above lncRNAs, exosomal MRPL23 antisense RNA 1 (MRPL23-AS1) derived from salivary adenoid cystic carcinoma (SACC), which increased microvascular permeability and promoted the metastasis of SACC to the lungs (139). Figure 3 shows that exosomal lncRNAs secreted by lung cancer cells are transmitted to target cells, which could regulate metastasis, immune response, EMT, and cancer-associated fibroblasts.

## Exosome-Derived LncRNAs in Lung Cancer Drug Resistance

Despite a variety of new antitumor drugs appearing every year, the cure rate and 5-year survival rate for lung cancer patients are still far from ideal. One important reason is that tumor cells quickly develop resistance once they are repeatedly exposed to the drugs. The latest research demonstrated that lncRNAs play an important role in lung cancer drug resistance. Colon cancer-associated transcript-1 (CCAT1) effectively sponged for miR-130a-3p and contributed to cisplatin resistance in NSCLC cells by targeting sex-determining region Y-box 4 (SOX4) (140). The deletion of lncRNA X inactivate-specific transcript (XIST) increased cisplatin chemosensitivity of NSCLC cells by inhibition of ATG7-mediated autophagy (141). Nuclear paraspeckle assembly transcript 1 (NEAT1) promoted paclitaxel resistance of NSCLC cells through enhancing caspase-3 expression and activating Akt/mTOR pathway (142).

The recent emerging exosome-derived lncRNAs are also important reasons for tumor cells acquiring drug resistance. Exosomal lncRNA activated in RCC with sunitinib resistance (lncARSR) was delivered to renal cell carcinoma to promote sunitinib resistance through sponging miR-34/miR-449, which further enhanced AXL and c-MET expression (15). A similar research was conducted that androgen-regulated transcript 1 (PART1) derived from exosomes facilitated gefitinib resistance *via* increasing Bcl-2 expression by competitively binding to miR-129 in ESCC cells (143). Exosomes separated from temozolomide-resistant glioblastoma (GBM) cells were full of SET-binding factor 2 (SBF2) antisense RNA1 (SBF2-AS1), which was the key factor to lead sensitive GBM cells turning to temozolomide (TMZ) resistance (144).

So far, four exosome-derived lncRNAs are confirmed to mediate drug resistance in lung cancer. One study finds that NSCLC cells exposed to gefitinib increases the expression of H19, which is delivered to other cells through exosomes secreted by primary tumor. Furthermore, exosomal H19 disseminates gefitinib resistance by targeting gefitinib-sensitive tumor cells (145). A similar study shows that H19 facilitates erlotinib resistance in NSCLC *via* miR-615-3p/ATG7 axis (146). Another exosome-mediated lncRNA in lung cancer is RP11-838N2.4, which facilitates erlotinib resistance in NSCLC (147). A series of experiments proved that exosomal RP11-838N2.4 is secreted from NSCLC cells of erlotinib resistance, and then transmitted to sensitive cells to promote erlotinib resistance. The third lung cancer-derived exosomal lncRNA is MSTRG.292666.16, which contributes to the acquired osimertinib resistance of lung cancer cells through regulating expression levels of miR-21, miR-125b, TGFβ, and ARF6 (148). The fourth exosomal lncRNA associated with drug resistance in lung cancer is urothelial carcinoma-associated 1 (UCA1), which facilitates gefitinib resistance in NSCLC by repressing miRNA-143 expression (149). These findings verify the mechanisms of exosome-derived lncRNAs regulating target therapy resistance and providing new insights for us to study drug resistance in lung cancer.

## Prospects of Exosome-Derived LncRNAs

Through the above literature review, we find that exosome-derived lncRNAs combine the advantages of exosomes and lncRNA characteristics, enabling lncRNAs to target distant cells or organs through the circulatory system. On the one hand, although many exosomal lncRNAs have been proven to be diagnostic markers for cancer, tumor-specific lncRNAs are still to be explored at present. Especially in the field of lung cancer research, the mechanism of exosome-derived lncRNAs in tumor progression is still in its infancy. On the other hand, the modification of exosomes and lncRNAs through biotechnology provides new ideas and insights for accurate evaluation of tumorigenesis and effective intervention in tumor progression. In addition, current studies have shown that lncRNA functions are far more complex than we know, and they may encode proteins (150, 151). Ahadi and colleagues found that there were miRNA seed sequences in lncRNAs, such as miR-18a, miR-93, and miR-106b (152). These exosome-derived lncRNAs also contained RNA and protein binding sites. This new finds may verify that exosomal lncRNAs are involved in tumorigenesis. The emerging exosome-wrapped lncRNAs would be the expectable method to cure lung cancer or at least detect lung cancer at its early stage to improve survival rate.

Considering the targeted delivery function of exosomes, cell biology, pharmaceutical science, and nanotechnology are attracted to the new era of human-derived nanovesicles. Aptamer-mediated drug delivery system is considered to be a prominent therapeutic for nanodelivery (87). For example, after fusing with anti-epithelial cell adhesion molecule cancer (EpCAM), aptamer could carry transferrin/aptamer across blood–brain barrier to cure brain diseases (153). Aptamer-targeted exosomal delivery is the embodiment of the combination of nanomedicine and exosome, which provide a much more optimized targeted drug delivery systems compared with traditional nanoparticle-based systems. With the help of nanoengineering technology, exosome-derived lncRNAs could be modified to carry a specific sequence or molecule that inhibits cancer cells, which will achieve a real sense of precision treatment for lung cancer.

## Conclusion

In this review, we briefly summarize the functional characteristics of exosomes and lncRNAs, expound the role of exosome-derived lncRNAs in tumors, especially in lung cancer, and look forward to the advantages of the combination of exosome-derived lncRNAs with nanomedicine in achieving targeted drug delivery and tumor precision treatment. At present, there are relatively few studies about exosome-derived lncRNAs in lung cancer, but more studies that can give us an in-depth understanding of their regulatory mechanism are under way. Although the functions of exosome-derived lncRNAs in tumors are similar, the tumorigenesis and progression of any cancer has its own unique rules and mechanisms. Currently, the mainstream research direction of exosome-derived lncRNAs is that they can be used as non-invasive tumor markers to diagnose tumors and as major gene targets for antitumor therapy. However, the pathophysiological mechanism of exosomal lncRNA in tumors still needs to be further studied. Meanwhile, under the current technical conditions, exosomal lncRNAs are still difficult to use as biomarkers due to their small number in body fluids and heterogeneity, which may cause false negatives or positives in cancer diagnosis. In future studies, more specific exosomal lncRNAs in different cancer should be discovered. With the combination of nanoengineering and molecular biology, exosome-mediated lncRNAs for precision nanomedicine will provide novel methods for cancer diagnosis and therapy.

## Author Contributions

TF wrote the manuscript. JH and NS revised and approved the manuscript. All authors contributed to the article and approved the submitted version.

## Conflict of Interest

The authors declare that the research was conducted in the absence of any commercial or financial relationships that could be construed as a potential conflict of interest.
